# Fine-Tuning the Details: Post-encoding Music Differentially Impacts General and Detailed Memory

**DOI:** 10.1523/JNEUROSCI.0158-25.2025

**Published:** 2025-06-23

**Authors:** Kayla R. Clark, Stephanie L. Leal

**Affiliations:** ^1^ Department of Psychological Sciences, Rice University, Houston, Texas 77005; ^2^ Department of Integrative Biology & Physiology, UCLA, Los Angeles, California 90095

**Keywords:** emotional arousal, interference, memory, music, pattern separation

## Abstract

Music can effectively induce emotional arousal, which is associated with the release of stress hormones that are important for the emotional modulation of memory. Thus, music may serve as a powerful modulator of memory and mood, making it a promising therapeutic tool for memory and mood disorders such as Alzheimer's disease or depression. However, music's impact on memory depends on its features, timing, and ability to elicit emotional arousal. In the current study, we manipulated various features of music played during post-encoding memory consolidation to elicit emotional arousal and impact subsequent memory in men and women. We found that larger increases and moderate decreases in post-encoding music-induced emotional arousal from baseline resulted in gist versus detail trade-offs in memory, with improved general memory but impaired detailed memory, while moderate increases in arousal from baseline corresponded to improved detailed memory, but impaired general memory. Importantly, relative to controls, music-induced emotional arousal demonstrated unique impacts on detailed memory that are crucial in supporting episodic memory. These findings suggest that music intervention does not uniformly impact memory and has important implications in developing personalized music-related interventions for those with memory and mood impairments.

## Significance Statement

Music may be a powerful tool for modulating memory and mood, offering therapeutic potential for disorders such as Alzheimer's disease and depression. We found that individual differences in emotional arousal following music exposure influenced both general memory and detailed memory performance. Compared with controls, music specifically impacted memory for details, highlighting its potential to target specific aspects of memory. These findings suggest that music interventions may not uniformly enhance memory, emphasizing the need for personalized approaches in treating memory and mood impairments.

## Introduction

Music is an integral part of everyday life—people often listen to it while completing routine tasks, exercising, socializing, or commuting. Given its constant presence, it is no surprise that music becomes entwined with our memories and influences how we process them (Jäncke, 2008). Music also serves as a cultural and social bridge, fostering shared experiences across individuals and communities (Cross, 2001). Listening to meaningful music frequently evokes memories, often accompanied by strong emotional components (Janata et al., 2007). One possible mechanism underlying music's impact on memory is its ability to elicit emotional responses. While emotional arousal from music seems to be a universal phenomenon, individual responses to music remain highly personal and subjective (Sloboda and Juslin, 2001; CH-Wang et al., 2022).

Several features of music influence emotional arousal in listeners, including emotional valence, familiarity, and the pleasure derived from listening (Ho and Loo, 2023). Negative stimuli tend to elicit stronger arousal than positive ones (Garavan et al., 2001), while enjoyment of and familiarity with stimuli enhance both pleasure and emotional response (Rickard, 2004; Van Den Bosch et al., 2013). These factors shape how effectively music induces emotional arousal and should be considered when evaluating its impact. While music shows promise as a therapeutic tool for improving mood and cognition in age-related and neuropsychiatric conditions, further research is needed to personalize its use for memory enhancement.

The timing of music intervention is crucial for its impact on behavior (Judde and Rickard, 2010). While studies have explored music played before, during, or after learning, separating music's effects from learning itself can be challenging (Rickard et al., 2005). Post-encoding emotional arousal designs address this issue more effectively (Cahill and Alkire, 2003; McGaugh, 2018). For instance, playing emotionally arousing music ∼20 min after learning has been shown to enhance word recall (Judde and Rickard, 2010). According to the emotional arousal hypothesis, such experiences trigger norepinephrine (NE) and cortisol release shortly after encoding, which modulate memory via the hippocampus and basolateral amygdala (BLA) (Cahill and McGaugh, 1995; McGaugh, 2004). Since music reliably induces emotional arousal (Juslin and Västfjäll, 2008), administering it during early memory consolidation may enhance memory and hippocampal function.

Notably, emotional arousal does not uniformly enhance memory. While it often improves post-encoding memory (McGaugh, 2018), it can favor gist-based over detailed information (Kensinger, 2009). This aligns with the Yerkes–Dodson law, which suggests cognitive performance peaks at moderate arousal but declines if arousal is too high or low (Yerkes and Dodson, 1908), as well as Wundt's theory linking optimal arousal to maximum pleasure (Wundt, 1904 ). Since music is closely tied to reward and pleasure (Ferreri and Rodrigues-Fornells, 2022), it may effectively induce optimal arousal. Music-induced dopamine and NE release may enhance memory consolidation by promoting hippocampal plasticity, including long-term potentiation (LTP; Huang and Kandel, 1995; Tully and Bolshakov, 2010; Leal et al., 2014).

One way to capture gist versus detail trade-offs of emotional memory is by applying a pattern separation framework. Two distinct hippocampal computations support gist and detailed memory, known as pattern completion and pattern separation, respectively (Yassa and Stark, 2011). Pattern completion is the process of reactivating a previous experience from a partial or degraded cue (generalization) and relies on hippocampal subfield CA3 (Treves and Rolls, 1994). In contrast, pattern separation is the process of orthogonalizing overlapping experiences as distinct representations, which relies on the dentate gyrus (DG) subregion of the hippocampus (Yassa and Stark, 2011). Mnemonic discrimination tasks (MDTs) are long-term episodic memory tasks that have been designed to behaviorally tax hippocampal pattern separation by including similar “lure” stimuli during the memory test (Stark et al., 2019). Emotional MDTs show that emotional gist is preserved while detail is often lost (Leal et al., 2014). In one study using post-encoding stress (Trier Social Stress Test), moderate cortisol levels improved negative mnemonic discrimination, while lower or higher levels impaired it, reflecting an inverted U-shaped relationship (Cunningham et al., 2018).

While music is effective at inducing emotional arousal (Juslin and Västfjäll, 2008), the specific features driving this effect remain unclear (Ho and Loo, 2023). Factors like familiarity, emotional valence, and pleasantness may influence arousal, but responses vary widely across individuals (CH-Wang et al., 2022). Given reported gist versus detail trade-offs in emotional memory, a more precise analysis of how music affects different aspects of memory is needed.

## Materials and Methods

### Experimental design

We systematically varied features of music administered in a post-encoding memory paradigm using an MDT that taxes hippocampal pattern separation to assess (1) whether post-encoding music may be an effective modulator of memory performance, (2) which extra-musical variables (e.g., valence, familiarity, etc.) influence music's ability to induce emotional arousal, and (3) whether music-induced emotional arousal uniformly influences memory (e.g., gist vs detail information). First, we hypothesized that music would effectively induce emotional arousal. We predicted that negative stimuli may more consistently induce emotional arousal as compared with positive stimuli (Garavan et al., 2001) and that familiarity may facilitate increases in music-induced emotional arousal (Salimpoor et al., 2009; Van Den Bosch et al., 2013). Second, we hypothesized that music-induced emotional arousal may lead to gist versus detail trade-offs in memory, such that general memory may be preserved while detailed memory may be impaired (Leal et al., 2014). However, there may be an optimal amount of arousal that is associated with this gist versus detail trade-off in memory in line with the Yerkes–Dodson law (Christianson, 1992).

### Participants

**Figure 1.**
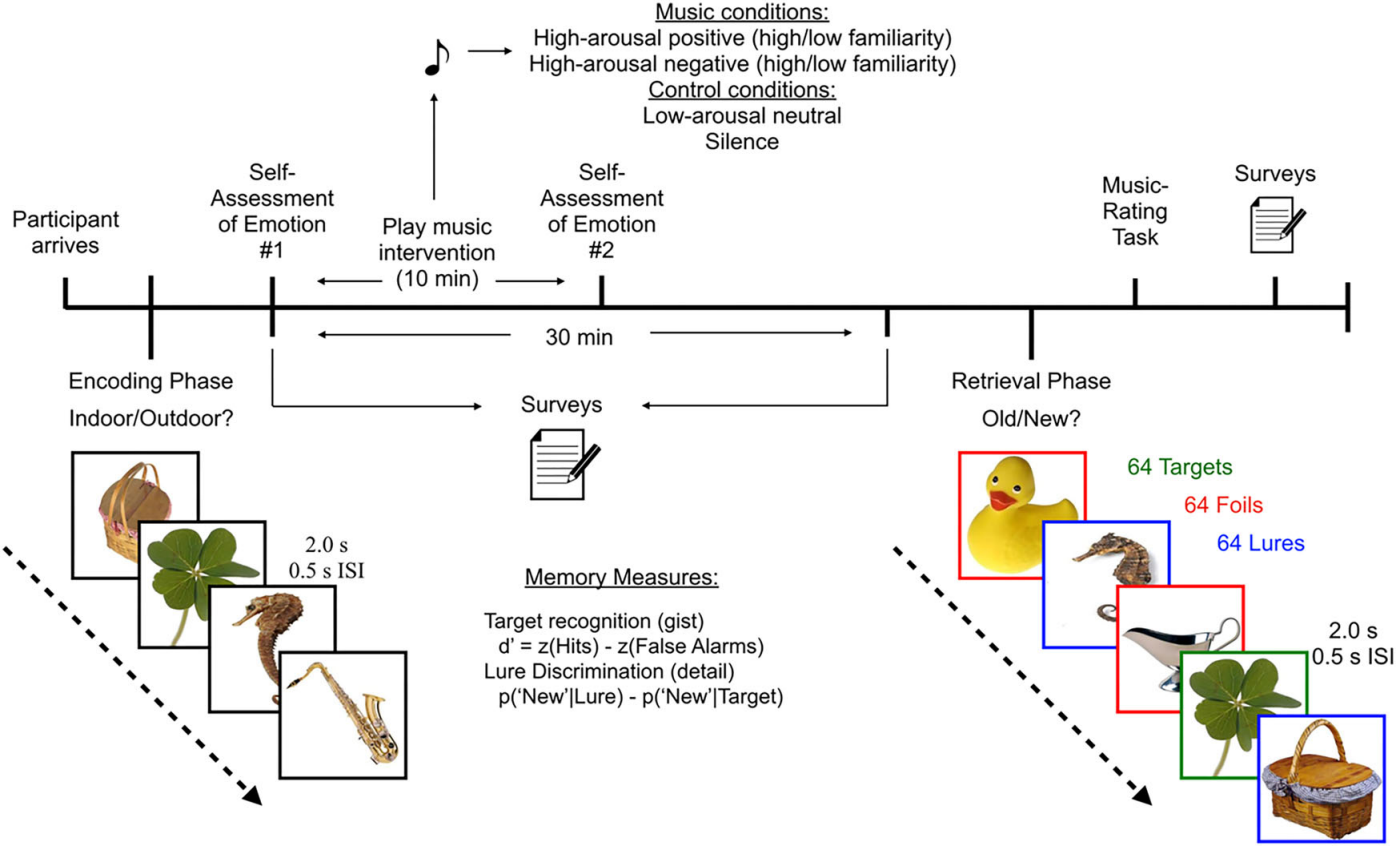
Study timeline and procedures. Participants completed the encoding phase of the object MDT, followed by a measure of current affective state (affect grid). Then, participants completed surveys for a 30 min period. During the first 10 min after encoding, participants listened to music, sound, or silence that corresponded to their assigned experimental condition. Following the 10 min music or sound intervention, participants completed another assessment of current affective state to assess changes in affect (arousal and valence) induced by the intervention and then continued completing questionnaires. Following the 30 min period, participants completed the retrieval phase of the MDT and completed the session by completing more surveys.

We enrolled 130 Rice University undergraduate students ages 18–35 in the study (see Table 1 for demographic information). Participants were compensated with course credits for psychology courses via SONA, and informed consent was obtained from all participants, with all procedures approved by the Rice University Institutional Review Board (IRB-FY2020-6: Cognitive, MRI, and PET studies of memory systems across the lifespan). Participants were randomly assigned into one of six conditions, which included four high-arousal music conditions that were stratified by emotional valence and familiarity [positive high familiarity (P-HF), negative high familiarity (N-HF), positive low familiarity (P-LF), negative low familiarity (N-LF)], one neutral sound condition that was not music (e.g., white noise, fireplace crackling; neutral valence, moderate familiarity), and one silent condition. Seven participants were removed due to missing data, outliers, or abnormalities during experimental sessions that may have affected data quality. One participant was an outlier when assessing change in arousal and memory performance (identified using the “bagplot” function in the “aplpack” package in R; Wolf et al., 2009), three participants were missing data, and three participants experienced anomalous events during music administration or inattention to instructions that resulted in poor data quality. Study timeline and procedure can be found in Figure 1.

**Table 1.** Participant demographics across experimental conditions

Variable	Positive high familiarity (*N* = 22)	Negative high familiarity (*N* = 23)	Positive low familiarity (*N* = 22)	Negative low familiarity (*N* = 19)	Neutral (*N* = 21)	Silent (*N* = 23)
Age (years)						
Mean	19.2	19.4	19.1	19.4	18.9	19.3
SD	1.11	1.23	0.99	1.07	1.09	1.06
Gender						
Male	8	10	8	8	11	9
Female	14	12	13	11	10	14
Non-binary	0	1	1	0	0	0
Race						
White	4	10	8	8	5	10
Asian	7	8	12	10	12	9
Black	5	2	1	0	2	2
Two or more races	4	3	1	1	2	1
Other	2	0	0	0	0	1

### Music and sound selection

Given that much of the previous research involving music and emotion or cognition utilized classical music (Eerola and Vuoskoski, 2013), we selected classical musical pieces that varied across our extra-musical features of interest and collected ratings in a separate sample of participants on emotional arousal, emotional valence, familiarity, and pleasantness (Tables S1-3, Fig. S1-2). Previous research has suggested that valence and familiarity of the music may have differential effects on the ability of music to induce emotional arousal and modulate cognition (Bullack et al., 2018). As such, we chose to match subjective ratings of emotional arousal across music conditions to more easily assess whether any subsequent differences manifest in emotional arousal or memory performance due to emotional valence or familiarity.

**Figure 2.**
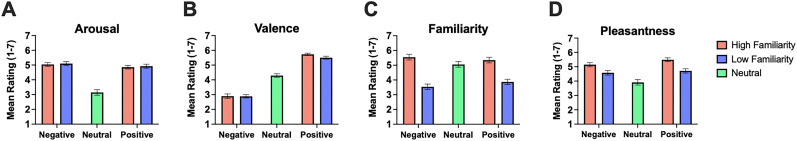
Matched music stimuli across arousal, valence, familiarity, and pleasantness dimensions. Negative and positive high- and low-familiarity music selections matched across levels of arousal (***A***), valence (***B***), familiarity (***C***), and pleasantness (***D***), with complementary levels of lower arousal, neutral valence, relative moderate familiarity, and lower pleasantness for neutral sound selections relative to music selections.

Musical stimuli were collected by searching keywords into internet search engines and media sites (e.g., YouTube) using emotional key words such as “exciting,” “calming,” “angry,” “happy,” “sad,” “joyful,” “scary,” and “classical.” We used item-wise matching to select three songs per condition (∼10 min of music) to have high levels of arousal and intentionally varied levels of emotional valence, familiarity, and pleasantness based on subjective ratings (Fig. 2). Item-wise matching allows for directly comparable sets of items across our designated dimensions. Thus, we were able to generate matched music stimuli within specified boundaries such that the set of three songs (e.g., N-HF) had similar ratings across emotional arousal (Fig. 2*A*), valence (Fig. 2*B*), familiarity (Fig. 2*C*), and pleasantness (Fig. 2*D*).

Item-wise matching was conducted using the “set_option”, “split_by”, “control_for”, and “generate” functions within the LexOPS package in R (Taylor et al., 2020), which allow the user to specify each of the parameters to be met or controlled for when generating matching sets of stimuli. Music samples were first organized into valence (positive and negative) and familiarity categories (high and low familiarity) based on previous Likert scale data. Positive music was rated above a 4 and negative music was rated below a 4 on a Likert scale (1–7, where 7 was extremely positive, 4 was neutral, and 1 was extremely negative). Familiar music was rated above a 4 and nonfamiliar music was rated below a 4 on a familiarity scale (1–7, where 7 was extremely familiar and 1 was not familiar at all). We used the LexOPS package in R (Taylor et al., 2020) to set parameters for matching music stimuli such that arousal ratings, familiarity ratings, and pleasantness ratings were as close to each other as possible across these groups. LexOPS produced three songs per valence-familiarity pair, so that we had three songs for each of the four valence-familiarity music categories. This resulted in the four music conditions included (P-HF, N-HF, P-LF, N-LF). All musical pieces were rated as highly arousing. Song selections for each condition can be found within Table S2. Musical features of the chosen songs (e.g., beats per minute, key, instrument composition, and song duration) can be found in Table S3. For more details on musical selection analyses, see Figure S1.

For the neutral, non-music sound condition, we selected both man-made and natural sounds, such as running water, ambient noise on an airplane, and the crackling of a fireplace (Fig. S1). Based on the item-wise matched values for arousal (Fig. 2*A*), valence (Fig. 2*B*), familiarity (Fig. 2*C*), and pleasantness (Fig. 2*D*) previously determined for the music stimuli, we chose three sounds (∼10 min of sound) that complemented the music pieces by using moderate levels of familiarity and pleasantness ratings, along with a neutral valence rating and a low level of arousal (Fig. 2*A–D*). Sound selections for this condition can be found in Figure S1 and Table S2. This neutral sound condition provides an “active” control such that participants experience auditory stimuli without receiving music-specific, arousal-based effects (Groarke and Hogan, 2019). Importantly, while the sounds are rated as “neutral” based on subjective ratings, the sound may not be absent of emotional cues, as neutral sounds may circumscribe an emotional state that does not necessarily fit into either “positive” or “negative” emotional states but is ambivalent, rather than devoid of emotion completely (Dellacherie et al., 2008).

### Mnemonic discrimination task

Once participants were assigned into one of the six experimental conditions discussed above, they engaged in a well-validated object MDT that has been shown to tax hippocampal pattern separation and consists of two phases (Stark et al., 2013). In the encoding phase, participants viewed 128 images of neutral, common household objects and were asked to classify the object as an “indoor” item or an “outdoor” item via button presses on a computer keyboard to allow for incidental encoding. Following a 30 min delay, participants completed the retrieval phase. During the retrieval phase, participants were presented with 192 images. These images were equally divided into images that were either identical to the encoding phase (targets), entirely new images (foils), or images that were similar to those presented during the encoding phase (lures). Participants were instructed to classify these images as either “Old” (the V key) or “New” (the N key), which allowed for the derivation of two memory measures: target recognition and lure discrimination. Target recognition is a measure of general memory for repeated images. Target recognition is measured by a discriminability index (*d*’), which is calculated as *z*(Hits) − *z*(False Alarms) and is corrected for response bias. Lure discrimination is a measure of detailed memory and is a behavioral correlate of pattern separation, as participants are required to discriminate between similar images and correctly identify a lure image as “New” and a previously shown target image as “Old.” Lure discrimination index (LDI) is calculated as *p*(“New”|Lure) − *p*(“New”|Target), which is corrected for response bias. All images in both the encoding and testing phases were displayed for 2 s with an ISI of 0.5 s on a white background.

### Music intervention during consolidation

Immediately following the encoding phase of the MDT, participants were provided with a pair of headphones and were played music, neutral sound, or silence that corresponded to their assigned condition for ∼10 min while completing questionnaires regarding their medical history, current levels of depression and anxiety, their personality, and emotion regulation tendencies (see Text S1 for complete descriptions of questionnaires). Each of the active auditory conditions consisted of three songs or neutral sounds. Song and sound clip order was randomized across participants within each experimental condition. Volume levels for listening were preset at a standard level; however, prior to beginning the music intervention, participants adjusted the volume up or down one level in accordance with their comfort. Following the 10 min of music, sound, or silence, participants continued completing questionnaires in silence for an additional 20 min to allow for emotional arousal to dissipate prior to completing the retrieval phase of the MDT. After completing retrieval, participants completed a ratings task akin to that used in the initial selection of stimuli where participants provided ratings of emotional arousal, emotional valence, familiarity, and pleasantness for the music or sound condition they were assigned to. Lastly, participants completed additional questionnaires regarding how they tend to engage with music and their musical expertise (Text S1).

### Measure of emotional arousal

To assess how emotional arousal changed in response to music listening, participants completed an affect grid (Russell et al., 1989), a single item assessment of both emotional valence and arousal immediately prior to and following the 10 min music or sound intervention period. Participants reported their subjective perception of their current emotional state by selecting a square on a 9 × 9 grid such that the *x*-axis represented emotional valence ranging from unpleasant (1) to pleasant (9), and the *y*-axis represented emotional arousal ranging from low arousal (1; e.g., depression, sleepiness, relaxation) to high arousal (9; e.g., stress, excitement). This allowed us to calculate changes in subjective levels of emotional arousal after music listening such that the baseline arousal level (prior to music or sound intervention) was subtracted from the arousal level following the music or sound intervention to produce a change in arousal score. We also calculated changes in subjective levels of emotional valence after music or sound listening such that the baseline valence level was subtracted from the post-music or sound listening valence level; however, this was not our primary measure of interest, and the following analyses will be focused on changes in emotional arousal given our hypotheses.

### Statistical analyses

Analyses were conducted using R studio version 2023.12.1+402. Planned analyses consisted of one-way ANOVAs and paired *t* tests or Welch's *t* test (two-tailed) where appropriate. Statistical values were considered significant at a final correct alpha of 0.05, which controlled for Type I error. Post hoc contrasts were conducted using Scheffé's method. To evaluate the strength of the reported relationships, we used Cohen's *d* and partial eta squared (*η*_p_^2^) for parametric analyses and eta squared (*η*_p_) and Wilcoxon effect size *r* for nonparametric analyses. An effect size of 0.2, 0.5, and 0.8 constitutes a small, medium, and large effect size, respectively, for Cohen's *d* (Cohen, 1988). An effect size of 0.02, 0.05, and 0.14 constitutes a small, medium, and large effects size, respectively, for partial eta squared. An effect size of 0.1, 0.3, and 0.5 constitutes a small, medium, and large effect size, respectively, for the Wilcoxon effect size *r* (Cohen, 1988). An effect size of 0.01, 0.06, and 0.14 constitute a small, medium, and large effect size, respectively, for eta squared. To assess the relationship between music-induced emotional arousal and memory performance, we conducted *k*-means clustering (MacQueen, 1967), a machine learning algorithm that groups observations into a preset number of clusters based on the nearest mean (i.e., cluster center or cluster centroid). Clusters may be based on one or more variables which have been standardized to allow groups of observations to aggregate based on similarity to one another. Each data point is allocated to a cluster based on reducing the in-cluster sum of squares, allowing data points to be assigned to the nearest cluster while also keeping the clusters as small as possible. Means produced from *k*-means clustering are the centroids of the respective number of clusters and represent the average of the data within the cluster.

To select the number of clusters used in analyses, we utilized the elbow method (Thorndike, 1953), whereby the sum of the squared distance between a data point and its respective centroid is plotted while varying the number of clusters from 1 to 10. As the number of clusters increases, the Within-Cluster Sum of Squares (WCSS) decreases, representing that the datapoints within a cluster are closer together. Importantly, as the number of clusters increases, there comes a point where the WCSS does not decrease drastically as the number of clusters continues to increase, effectively creating a distinctive bend or “elbow” in the graph. Such a point is thought to indicate that the number of clusters at this elbow is the optimal number of clusters to use for analyses within a dataset, as additional clusters likely will not reveal additional useful information about the data.

Once the clusters were established, prior to conducting analyses, we assessed normality and homogeneity of cluster groups. All clusters created as a result of *k*-means clustering were homogeneous; however, some groups displayed non-normal distribution of the data. As such we used the appropriate nonparametric tests when needed in the following analyses. Once assumptions were assessed, we compared the mean change in arousal across the cluster groupings separately for music participants and control participants using one-way ANOVAs and *t* tests, respectively (Table S4).

### Transparency and openness

All data have been made publicly available on GitHub (https://github.com/lealmemorylab/music). This study was not preregistered.

## Results

### Music effectively increased emotional arousal

**Figure 3.**
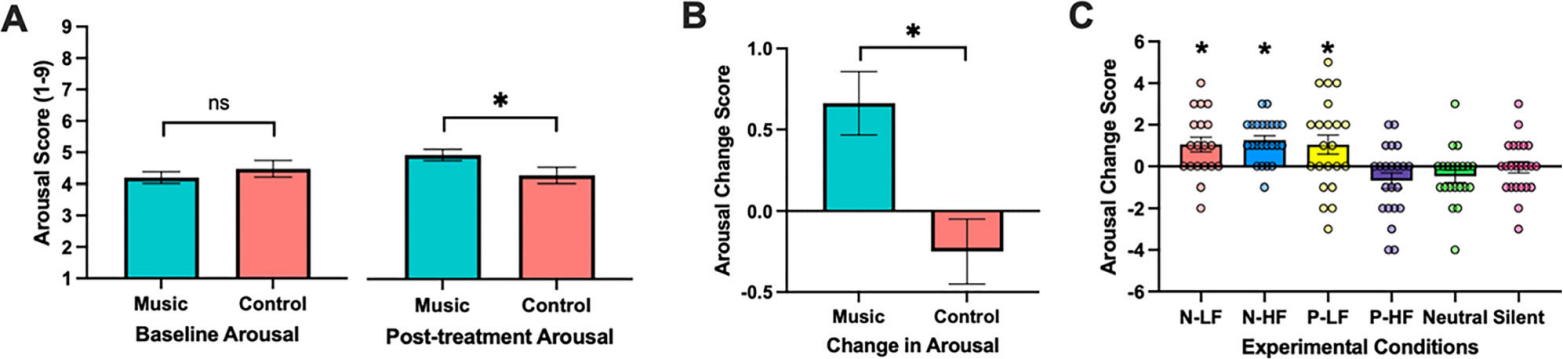
Arousal scores in music and control conditions. ***A***, Baseline and posttreatment levels of arousal as measured via the affect grid (1–9) in music and control conditions. ***B***, Change in arousal via the affect grid (posttreatment – baseline) in music and control conditions. ***C***, Change in arousal across all six experimental conditions [Negative Low Familiarity (N-LF), Negative High Familiarity (N-HF), Positive Low Familiarity (P-LF), Positive High Familiarity (P-HF), Neutral, and Silent].

To assess whether the music intervention was effective in inducing emotional arousal, we collapsed across the four music conditions and across the two control conditions and assessed differences in baseline levels of arousal, posttreatment levels of arousal, and changes in emotional arousal using scores from the affect grid. One-way ANOVAs revealed no differences in mean levels of arousal between music and control groups at baseline (*t*_(86)_ = −0.86, *p* = 0.39, d = −0.16; Fig. 3*A*), and significant differences between mean levels of arousal between music and control groups at posttreatment (*t*_(86)_ = 2.03, *p* = 0.04, d = 0.37; Fig. 3*A*) and change in arousal (*F*_(1,128)_ = 8.73, *p* = 0.004, *η*_p_^2^ = 0.06; Fig. 3*B*) such that music conditions demonstrated greater levels of arousal posttreatment and greater changes in arousal from baseline to posttreatment, respectively. Such results show that music was effective in inducing emotional arousal in listeners as compared with control conditions.

Next, we examined arousal effects across each experimental condition. Reflecting the pattern found in the collapsed music and control groups, there were no differences among the six conditions in their mean baseline levels of arousal prior to the post-encoding manipulation (*F*_(6,124)_ = 0.34, *p* = 0.89, *η*_p_^2^ = 0.01), but there were significant differences among the six conditions in their posttreatment levels of arousal (*F*_(6,124)_ = 4.08, *p* = 0.002, *η*_p_^2^ = 0.14) and their levels of change in arousal (*F*_(6,124)_ = 6.63, *p* < 0.001, *η*_p_^2^ = 0.21; Fig. 3*C*). N-HF, P-LF, and N-LF conditions exhibited increases in arousal relative to Silent, Neutral, and P-HF (*p* < 0.001). However, it is important to note that there were individual differences in experiencing a change in arousal from listening to music, in which not all participants showed increased arousal. In fact, some showed a decrease in arousal (Fig. 3*C*). Therefore, people did not consistently show increased arousal in response to the music conditions even though all music conditions were subjectively rated as highly arousing (Fig. 2). We also collected ratings of the extra-musical features (valence, arousal, familiarity, pleasantness) of the music and sound conditions following the retrieval phase of the MDT as we did in the pilot ratings study and replicated ratings for emotional valence, emotional arousal, familiarity, and pleasantness across samples (Fig. S2).

### No differences in memory performance across experimental groups

**Figure 4.**
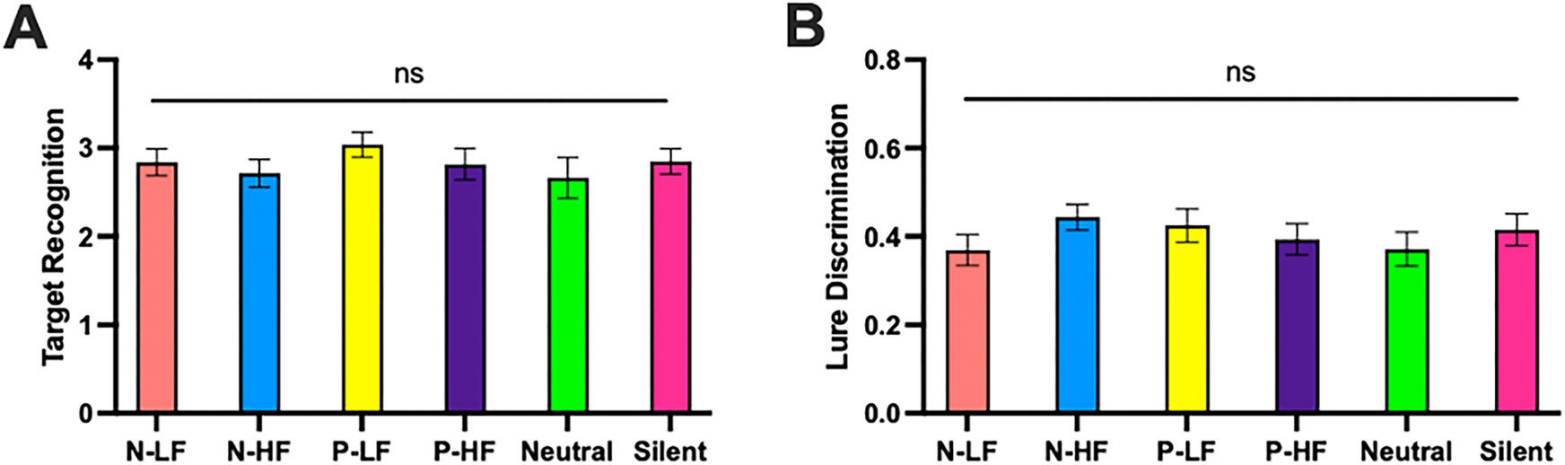
Memory performance across all music and control experimental conditions. ***A***, Target recognition (*d*’) scores across all six experimental conditions. ***B***, Lure discrimination index (LDI) scores across all six experimental conditions.

Despite the variability in emotional responses to each of the experimental conditions, music on average induced greater levels of emotional arousal. To test differences in memory across experimental groups, we utilized one-way ANOVAs for both target recognition and lure discrimination to assess how experimental condition impacted memory. There were no significant differences in memory across experimental groups for either target recognition (*F*_(5,124)_ = 0.61, *p* = 0.696, *η*_p_^2^ = 0.02; Fig. 4*A*) or lure discrimination (*F*_(5,124)_ = 0.72, *p* = 0.610, *η*_p_^2^ = 0.03; Fig. 4*B*). Importantly, given that one of the music conditions appeared to ineffectively induce emotional arousal, we also assessed whether the groups that effectively induced emotional arousal (N-LF, N-HF, P-LF) demonstrated improved memory performance relative to the control groups (Neutral, Silent). One-way ANOVAs revealed no differences in memory performance for either target recognition (*F*_(1,106)_ = 0.46, *p* = 0.498, *η*_p_^2^ = 0.00; Fig. 4*A*) or lure discrimination (*F*_(1,106)_ = 0.43, *p* = 0.515, *η*_p_^2^ = 0.00; Fig. 4*B*). Additional pairwise *t* tests between different conditions were all insignificant.

### Individual differences in music-induced emotional arousal

Given the individual differences we observed in emotional arousal across the various music conditions and our hypothesis that music-induced emotional arousal may be a strong modulator of memory, we aimed to examine change in arousal across all of the music conditions and across control participants using *k*-means clustering (see Materials and Methods for more information). We hypothesized that participants may have experienced varying levels of change in arousal in line with the Yerkes–Dodson law, which may differentially impact cognition. As such, participants may have experienced low, moderate, or high emotional arousal in response to music listening, where higher and lower levels of emotional arousal may demonstrate similar cognitive performance.

First, we utilized the elbow method to determine the appropriate number of clusters to use in *k*-means clustering based on change in arousal scores (Thorndike, 1953) of our music (P-HF, N-HF, P-LF, N-LF) and control (Neutral, Silent) participants (see Materials and Methods for details). We conducted these analyses separately in our music and control participants to more clearly understand how music-induced arousal may be impacting memory performance as compared with general non-music-induced arousal. Within our music participants (*N* = 86), there was a distinct bend, or “elbow” at three clusters (Fig. S3*A*), indicating that the data tended to fall into three clusters when assessing their levels of music-induced change in arousal. Within our control participants (*N* = 44), there was a distinct elbow at two clusters (Fig. S3*B*), indicating that the participants tended to fall into two clusters when assessing their levels of change in non-music-induced arousal. Based on the results from the elbow method examining change in arousal scores alone, we then conducted *k*-means clustering using three clusters for music participants (Fig. S3*C,D*) and two clusters for control participants (Fig. S3*E,F*) utilizing two variables in each instance of clustering: (1) change in arousal in conjunction with target recognition scores and (2) change in arousal in conjunction with lure discrimination scores. Given that each memory measure is tapping different aspects of episodic memory, this approach allowed us to examine any differential or specific interactions between change in arousal and each memory measure.

**Figure 5.**
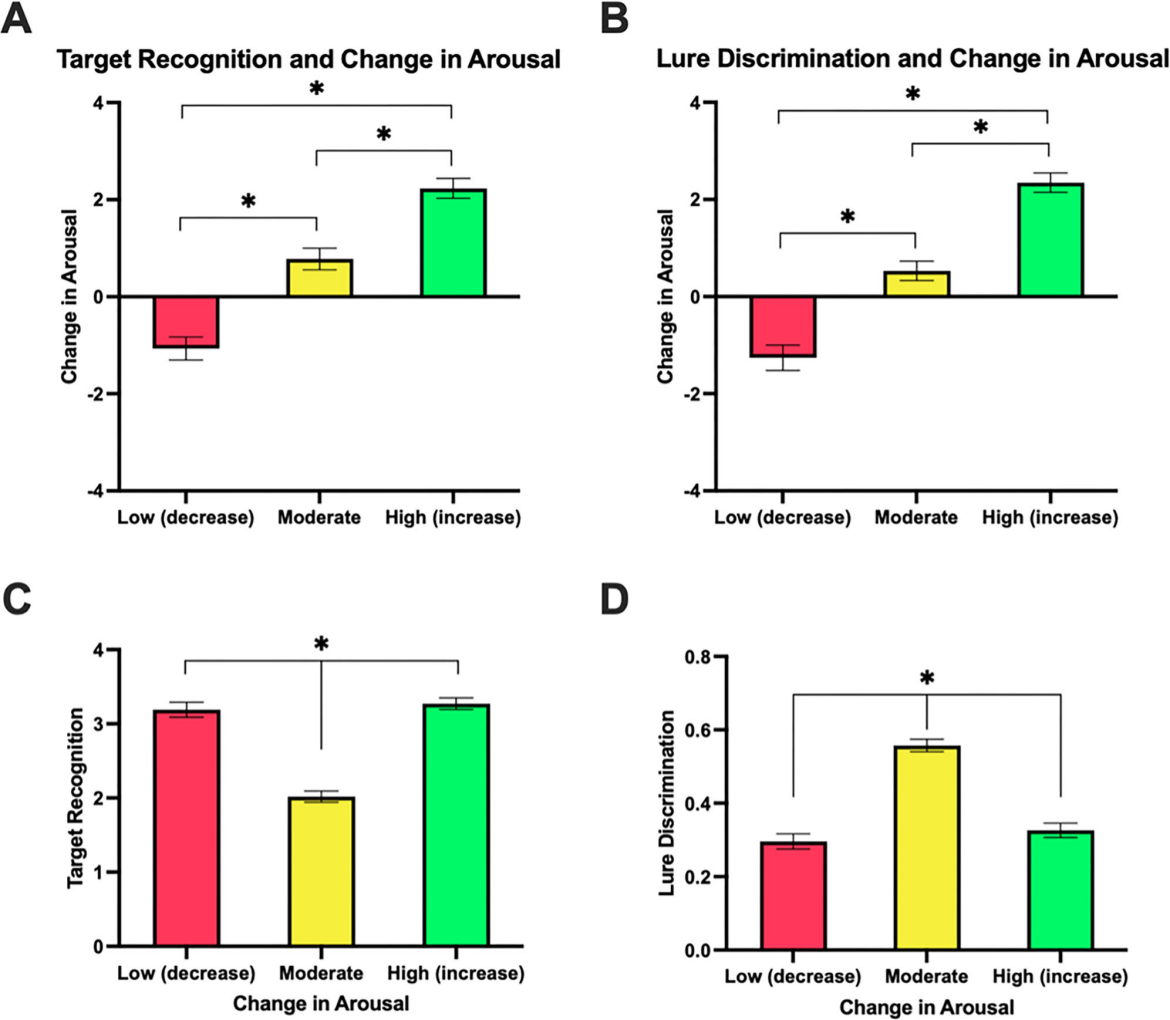
Change in arousal means and memory performance means for Low (decrease) change in arousal, Moderate change in arousal, and High (increase) change in arousal for each set of *k*-means clustering parameters in music participants. ***A***, Change in arousal means for clusters based on target recognition (*d*’) and change in arousal. ***B***, Change in arousal means for clusters based on lure discrimination and change in arousal. ***C***, Mean target recognition scores(*d*’) for clusters based on target recognition and change in arousal. ***D***, Mean lure discrimination scores for clusters based on lure discrimination and change in arousal.

For music participants, the change in arousal data in each of the clusters was not normally distributed. As such, we used the Kruskal–Wallis H test to confirm that there were significant differences in change in arousal means across clusters [music conditions clustered by change in arousal and target recognition: *H*(2) = 55.04, *p* < 0.001, *η*^2^ = 0.64 (Fig. 5*A*); music conditions clustered by change in arousal and lure discrimination: *H*(2) = 57.02, *p* < 0.001, *η*^2^ = 0.66 (Fig. 5*B*)]. More targeted independent two-group Wilcoxon–Mann–Whitney tests reveal that one cluster of participants experienced a decrease in emotional arousal (low arousal–decrease; *N* = 29 target recognition; *N* = 23 lure discrimination), one cluster experienced a moderate increase in emotional arousal (moderate arousal; *N* = 27 target recognition; *N* = 34 lure discrimination), and one cluster experienced a relatively strong increase in emotional arousal (high arousal–increase; *N* = 30 target recognition; *N* = 29 lure discrimination) where each of these groups means were significantly different from one another. This was the case for both the change in arousal in conjunction with target recognition clusters (Low vs Moderate: *z* = −4.77, *p* < 0.001, *r* = 0.64; High vs Moderate: *z* = 4.04, *p* < 0.001, *r* = 0.54; High vs Low: *z* = 6.68, *p* < 0.001, *r* = 0.87; Fig. 5*A*) and the change in arousal in conjunction with lure discrimination clusters (Low vs Moderate: *z* = −4.83, *p* < 0.001, *r* = 0.64; High vs Moderate: *z* = 5.33, *p* < 0.001, *r* = 0.67; High vs Low: *z* = 6.22, *p* < 0.001, *r* = 0.86; Fig. 5*B*).

**Figure 6.**
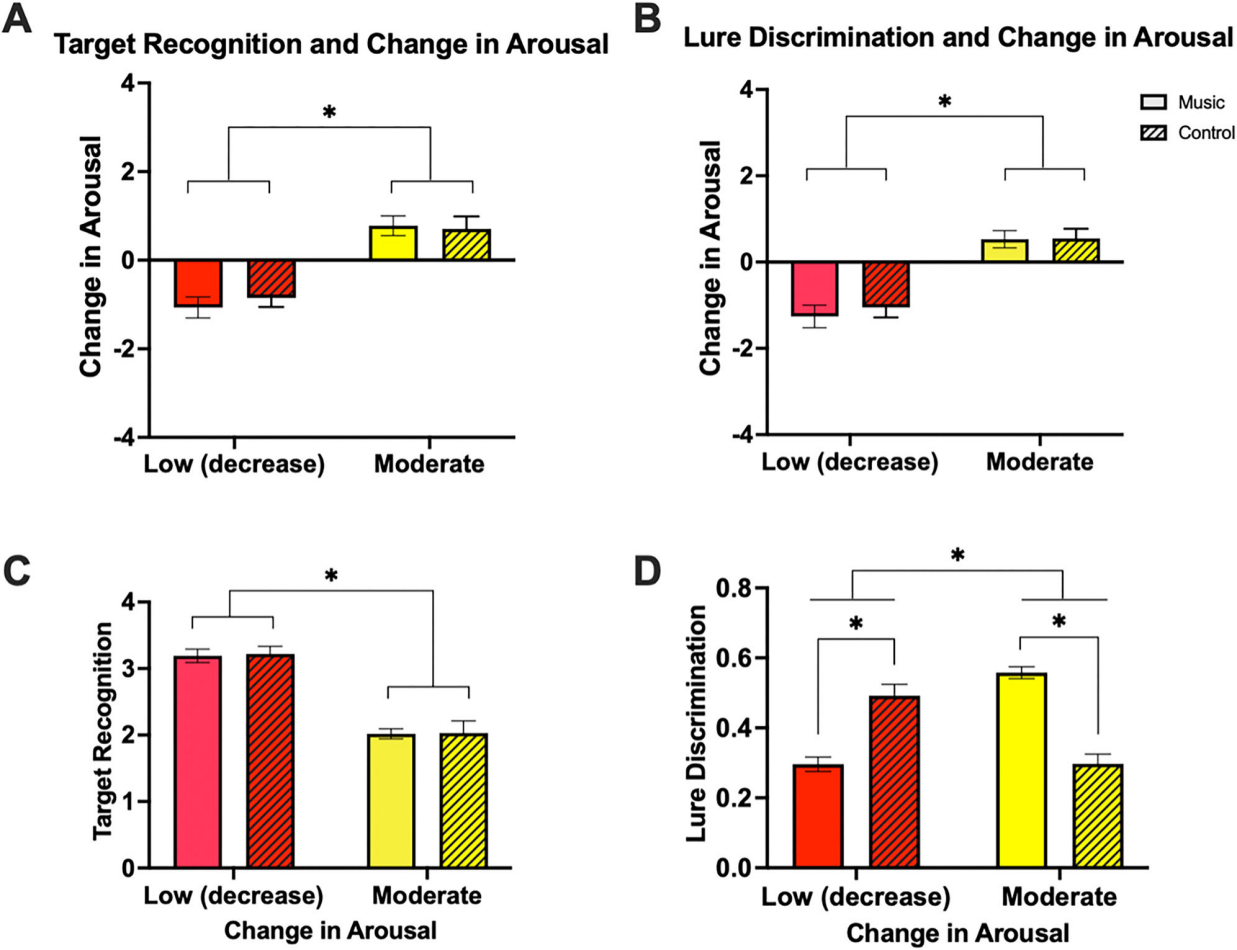
Change in arousal means and memory performance means for Low (decrease) change in arousal and Moderate change in arousal for each set of *k*-means clustering parameters in music participants (solid bars) and control participants (striped bars). ***A***, Change in arousal means for clusters based on target recognition (*d*’) and change in arousal. ***B***, Change in arousal means for clusters based on lure discrimination and change in arousal. ***C***, Mean target recognition scores (*d*’) for clusters based on target recognition and change in arousal. ***D***, Mean lure discrimination scores for clusters based on lure discrimination and change in arousal.

For control participants, the change in arousal data in each of the clusters was not normally distributed. As such, we used the independent two-group Wilcoxon–Mann–Whitney test to confirm that there were significant differences in change in arousal means across the two clusters (control participants clustered by change in arousal and target recognition: *z* = 3.95, *p* < 0.001, *r* = 0.60, Fig. 6*A*; control participants clustered by change in arousal and lure discrimination: *z* = 4.34, *p* < 0.001, *r* = 0.65, Fig. 6*B*). These tests revealed that control participants fell into one cluster that experienced a moderate decrease in emotional arousal (low arousal–decrease; *N* = 17 target recognition; *N* = 22 lure discrimination) and another cluster that experienced a moderate increase in emotional arousal (moderate arousal; *N* = 27 target recognition; *N* = 22 lure discrimination).

To compare levels of change in arousal across clusters between music and control participants, we utilized the Kruskal–Wallis H test due to non-normally distributed data within clusters. Specifically, we compared the Low (decrease) and Moderate clusters between music and control participants because these two clusters were consistently found across both music and control participants, where it is important to establish whether music and control conditions produced similar levels of change in arousal in each listening condition. Results showed significant differences in change in arousal means across clusters [music participants clustered by change in arousal and target recognition: *H*(2) = 38.75, *p* < 0.001, *η*^2^ = 0.39 (Fig. 6*A*); music participants clustered by change in arousal and lure discrimination: *H*(2) = 42.39, *p* < 0.001, *η*^2^ = 0.42 (Fig. 6*B*)]. More targeted independent two-group Wilcoxon–Mann–Whitney tests revealed that the Low change in arousal clusters were significantly different from the Moderate change in arousal clusters across both music and control participants. This was true for both the change in arousal in conjunction with target recognition clusters (music Low vs Moderate: *z* = 4.77, *p* < 0.001, *r* = 0.64; control Low vs Moderate: *z* = 3.95, *p* < 0.001, *r* = 0.60; Fig. 6*A*) and the change in arousal in conjunction with lure discrimination clusters (music Low vs Moderate: *z* = 4.83, *p* < 0.001, *r* = 0.64; control Low vs Moderate: *z* = 4.34, *p* < 0.001, *r* = 0.65; Fig. 6*B*). Conversely, when comparing the two Low and Moderate change in arousal clusters between music and control participants, we saw no differences in levels of change in arousal when the clusters were based on change in arousal in conjunction with target recognition (music Low vs control Low: *z* = −0.35, *p* = 0.735, *r* = 0.05; music Moderate vs control Moderate: *z* = 0.45, *p* = 0.662, *r* = 0.07; Fig. 6*A*), nor when the clusters were based on change in arousal in conjunction with lure discrimination (music Low vs control Low: *z* = −0.44, *p* = 0.669, *r* = 0.07; music Moderate vs control Moderate: *z* = 0.47, *p* = 0.642, *r* = 0.06; Fig. 6*B*).

### Opposing effects of music-induced emotional arousal on memory specificity

Next, we examined how memory performance varied among music participants in low, moderate, and high arousal clusters. For target recognition, we conducted a one-way ANOVA across change in arousal clusters (low, moderate, and high), which revealed a significant effect of arousal cluster (*F*_(2,83)_ = 64.88, *p* < 0.001, *η*_p_^2^ = 0.61; Fig. 5*C*), where those who experienced low or high music-induced emotional arousal demonstrated better target recognition compared with those who experienced moderate music-induced emotional arousal as revealed by post hoc Scheffe's tests (High vs Moderate: *t*_(83)_ = 10.29, *p* < 0.001, *d* = 2.73; Low vs Moderate: *t*_(83)_ = −9.54, *p* < 0.001, *d* = −2.55). Thus, those who exhibited a larger increase or a moderate decrease in music-induced emotional arousal from baseline, showed better target recognition compared with those who experienced moderate increases in arousal.

For lure discrimination, we conducted a one-way ANOVA across change in arousal clusters (low, moderate, and high), which revealed a significant effect of arousal cluster (*F*_(2,83)_ = 59.63, *p* < 0.001, *η*_p_^2^ = 0.59; Fig. 5*D*), where we found the opposite effect compared with target recognition performance—those who experienced low or high music-induced emotional arousal demonstrated worse lure discrimination compared with those who experienced moderate music-induced emotional arousal as revealed by post hoc Scheffe's tests (High vs Moderate: *t*_(83)_ = 4.88, *p* < 0.001, *d* = 1.29; Low vs Moderate: *t*_(83)_ = −4.84, *p* < 0.001, *d* = −1.30). Thus, those who exhibited a larger increase or a moderate decrease in music-induced arousal from baseline, showed worse lure discrimination compared with those who experienced moderate increases in arousal.

### Music-induced emotional arousal shows unique impact on lure discrimination compared with controls

Next, we examined how memory performance varied among control participants in low and moderate arousal clusters. For target recognition, there was a significant difference in memory performance between arousal clusters (*t*_(42)_ = −5.89, *p* < 0.001, *d* = −1.82; Fig. 6*C*), such that those who experienced a decrease in arousal demonstrated better target recognition compared with those who experienced an increase in arousal in line with those who listened to music. For lure discrimination, we found a significant difference between the two arousal clusters (*t*_(42)_ = −4.57, *p* < 0.001, *d* = −1.38; Fig. 6*D*), such that those who experienced a decrease in arousal demonstrated improved lure discrimination performance relative to those who experienced a moderate increase in arousal, in contrast to those who listened to music.

We then directly compared control and music participants across the Low and Moderate cluster groupings based upon similar mean levels of change in arousal in each cluster. As such, we utilized two-way ANOVAs across these four clusters. For target recognition, we conducted a two-way ANOVA with listening condition (music, control) and arousal cluster (Low, Moderate) as factors, which revealed a significant effect of arousal cluster (*F*_(1,96)_ = 108.44, *p* < 0.001, *η*_p_^2^ = 5.30; Fig. 6*C*), but no effect of listening condition (*F*_(1,96)_ = 0.04, *p* = 0.849, *η*_p_^2^ = 0.00; Fig. 6*C*), or interaction (*F*_(1,96)_ = 0.01, *p* = 0.928, *η*_p_^2^ = 0.00; Fig. 6*C*), suggesting music and control groups were performing similarly on target recognition. For lure discrimination, the same ANOVA revealed a significant effect of arousal cluster (*F*_(1,97)_ = 5.97, *p* = 0.016, *η*_p_^2^ = 0.05; Fig. 6*D*), a significant effect of listening condition (*F*_(1,97)_ = 4.68, *p* = 0.033, *η*_p_^2^ = 0.06; Fig. 6*D*), and an interaction between arousal cluster and listening condition (*F*_(1,97)_ = 89.03, *p* < 0.001, *η*_p_^2^ = 0.48; Fig. 6*D*). More targeted post hoc Scheffe's tests revealed that music and control participants demonstrated opposite patterns of performance, whereby music participants who experienced a moderate decrease in arousal demonstrated worse lure discrimination relative to control participants (music Low vs control Low: *t*_(43)_ = 5.49, *p* < 0.001, *d* = −1.64; Fig. 6*D*), and music participants who experienced a moderate increase in arousal demonstrated better lure discrimination relative to control participants (music Moderate vs control Moderate: *t*_(54)_ = −7.97, *p* < 0.001, *d* = 2.18; Fig. 6*D*). There were no significant differences between the low arousal control cluster and the moderate arousal music cluster (music Moderate vs control Low: *t*_(43)_ = −2.02, *p* = 0.258, *d* = 0.55; music Low vs control Moderate; *t*_(54)_ = −0.032, *p* = 1.00, *d* = −0.01; Fig. 6*D*).

## Discussion

This study examined how post-encoding music-induced emotional arousal affects memory by manipulating extra-musical variables such as valence, familiarity, and arousal. Overall, music modulated both general and detailed memory, but individual differences in emotional responses were crucial—participants listened to the same music yet responded differently. Compared with control groups, music significantly increased arousal, particularly for negative music regardless of familiarity, and for novel positive music. These findings align with prior research linking high-arousal music to increased autonomic activity (Khalfa et al., 2008; Lundqvist et al., 2009), though familiarity may moderate this effect. Furthermore, one music piece, *Radetzky March*, had viral popularity on social media, potentially distracting participants. Regardless, there was a significant amount of variability in reported emotional responses across all conditions, emphasizing the role of individual differences in emotional arousal induction. Despite increased arousal in music conditions, no group-level differences in memory performance were found. This led us to analyze memory in relation to individual arousal responses, rather than by condition alone, highlighting the importance of personalized emotional engagement.

*K*-means clustering revealed three distinct emotional response groups among music participants: those with large arousal increases, moderate increases, or moderate decreases. In the control group, two response patterns emerged: moderate increases or decreases. This variability highlights that while music generally induced arousal, responses were not uniform, emphasizing the importance of individual differences in music interventions. Such variability may stem from personal music preferences, musical features, or individual differences in limbic reactivity or “cortical arousability”—the brain's capacity to shift from low to high activity in response to stimuli (Coren and Mah, 1993; Kehoe et al., 2012). Future neuroimaging studies could explore the mesolimbic dopamine system to better understand individual differences in music reward sensitivity (Ferreri and Rodriguez-Fornells, 2022). Lastly, while self-report measures capture subjective emotional experience (Lazarus, 1991), incorporating physiological measures would offer more objective insights into emotional arousal.

Using an individual differences approach, we examined how changes in music-induced emotional arousal affected general memory (target recognition) and detailed memory (lure discrimination, taxing pattern separation). Listeners with either large increases or moderate decreases in arousal performed better on target recognition, while those with moderate arousal increases performed worse. In contrast, moderate arousal increases led to better lure discrimination, while large increases or moderate decreases impaired it. These results suggest arousal's impact on memory is not linear; instead, both increases and decreases can enhance memory depending on the measure—supporting a quadratic, inverted U pattern consistent with the Yerkes–Dodson law (Diamond et al., 2007).

Second, music-induced emotional arousal has differential impacts on memory that depend on how memory is measured, such that moderate increases in arousal improved lure discrimination but impaired target recognition. Notably, music-induced arousal improved lure discrimination compared with control-induced arousal, indicating music may better target memory processes like pattern separation (Yassa and Stark, 2011). These findings align with prior work showing optimal arousal enhances cognition (Christianson, 1992; Cahill et al., 2003) and that extremes in arousal, such as stress or anxiety, impair it (Wolf et al., 2016). Our results suggest a nuanced model: different arousal levels may benefit different memory processes—moderate arousal boosts detail memory, while greater or lesser arousal supports gist memory.

MDTs are sensitive to memory dysfunction compared with traditional episodic memory tasks (Stark et al., 2019). Our findings align with prior research showing better lure discrimination for neutral versus emotional stimuli both immediately and after 24 h (Leal et al., 2014), consistent with the Arousal-Biased Competition model (Mather and Sutherland, 2011), which suggests higher arousal amplifies gist-detail trade-offs, while moderate arousal reduces them. Future high-resolution neuroimaging studies could clarify the neural mechanisms behind these effects. We hypothesize that moderate music-induced arousal may enhance DG/CA3-BLA connectivity, supporting better lure discrimination, while greater increases or decreases in arousal may weaken this network. Music-related regions such as the auditory cortex, supratemporal gyrus, nucleus accumbens, ventral tegmental area, or orbitofrontal cortex may also modulate these effects (Blood and Zatorre, 2001; Karmonik et al., 2016; Reybrouck et al., 2018).

Importantly, the inversion of arousal-based memory effects was only observed in music listeners. Control participants exposed to non-music stimuli or silence did not show a gist versus detail trade-off, as their performance on target recognition and lure discrimination was similar. However, music-induced arousal specifically enhanced lure discrimination, suggesting that music may uniquely engage pattern separation processes crucial for episodic memory (Yassa and Stark, 2011). Even with similar arousal levels, music-induced arousal may offer a distinct mechanism compared with non-music arousal, warranting further exploration.

The change in arousal relative to baseline was similar for the decreased and moderate arousal groups across both music and control participants, with higher increases in arousal only observed in music listeners. Given we systematically chose high arousal music stimuli, we generally expected participants to show increased levels of arousal. Indeed, most participants did show increases in arousal when listening to music. While we observed a greater magnitude of increased arousal compared with the magnitude of decreased arousal (relative to baseline), we observed similar memory performance across these arousal conditions. However, when considering these three arousal groups in line with the Yerkes–Dodson law, participants showed a similar magnitude in increased and decreased arousal relative to moderate or “optimal” levels of arousal, rather than relative to baseline. Thus, the magnitude relative to “optimal” arousal rather than baseline may be a better indicator of the impact arousal will have on memory performance.

Timing of music intervention is crucial for understanding how music modulates memory, particularly during post-encoding consolidation when newly learned information is vulnerable to stress hormones impacting the hippocampus and BLA (Cahill and McGaugh, 1995; McGaugh, 2004). We found that music intervention shortly after encoding significantly impacted memory when tested after a short delay (∼30 min), but effects were only observed after taking an individual differences approach. Longer delays (e.g., 24 h) might exaggerate emotion's effects on memory (LaBar and Cabeza, 2006; Mather and Sutherland, 2011). Given that NE triggers rapid protein synthesis-dependent LTP essential for long-term memory (Tully and Bolshakov, 2010), future studies should assess memory at various intervals to evaluate lasting effects on memory consolidation (Hamann, 2001).

Several limitations exist in this study. We used classical music, commonly used in Western research, but individual music preferences may drive differences in arousal responses (Schäfer and Sedlmeier, 2010). Arousal predisposition (Coren, 1988) and age-related preferences (e.g., older adults preferring low-arousal music) may also influence music effectiveness (Kessler and Staudinger, 2009; Cohrdes et al., 2017). Since music is often used for affect regulation (Juslin and Laukka, 2004), individual preferences could impact how music-induced arousal modulates memory. To address these limitations, future studies could allow participants to select their preferred music, optimizing hedonic value and expanding the range of music, including non-Western selections. This approach aligns with Berlyne’s Theory (1971), suggesting that peak hedonic experiences lead to optimal arousal levels.

By assessing music's effects on emotion and memory processes that form our perception of the world, we may be better able to understand how music may be used as a multifaceted tool to improve mood and cognition across the lifespan and using an individualized approach. Future research should further examine how music perception may vary across different populations, ages, and cultures, and specifically how this perception influences music-induced arousal and subsequent effects on cognition, as this has implications for music as a tool for memory interventions across such populations.
